# A Phantom Study on Target Localization Accuracy Using Cone-Beam Computed Tomography

**DOI:** 10.4137/cmo.s808

**Published:** 2008-08-18

**Authors:** Hui Yan, Liwei Zhang, Fang-Fang Yin

**Affiliations:** Department of Radiation Oncology, Duke University Medical Center, Durham, NC 27710, U.S.A

**Keywords:** target localization, cone-beam computed tomography, on-board imager

## Abstract

The purpose of this study is to evaluate the 3-dimensional target localization accuracy of cone-beam computed tomography (CBCT) using an on-board imager (OBI). An anthropomorphic pelvis phantom was used to simulate a range of offsets in the three translational directions and rotations around each of the three axes. After a translational or rotational offset was applied, a CBCT scan of the phantom was followed by image registration to detect the offsets in six degrees. The detected offsets were compared to the offset actually applied to give the detection error of the phantom position. Afterwards, the phantom was positioned by automatically moving the couch based on the detected offsets. A second CBCT scan followed by image registration was performed to give the residual error of the phantom positioning. On the average the detection errors and their standard deviations along the lateral, longitudinal and vertical axis are 0.3 ± 0.1, 0.3 ± 0.1 and 0.4 ± 0.1 mm respectively with respect to translational shifts ranging from 0 to 10 mm. The corresponding residual errors after positioning are 0.3 ± 0.1, 0.5 ± 0.1 and 0.3 ± 0.1 mm. For simulated rotational shifts ranging from 0 to 5 degrees, the average detection error and their standard deviation around lateral, longitudinal, and vertical axes are 0.1 ± 0.0, 0.2 ± 0.0, and 0.2 ± 0.0 degrees respectively. The residual errors after positioning are 0.4 ± 0.1, 0.6 ± 0.1, and 0.3 ± 0.1 mm along the lateral, longitudinal and vertical directions. These results indicate that target localization based on CBCT is capable of achieving sub-millimeter accuracy.

## Introduction

The goal of external beam radiation therapy is to deliver a sufficient dose to the tumor volume while keeping dose to normal tissue below tolerance levels. Achieving this goal depends strongly on the precision in which radiation is delivered. The ultimate limitation is the geometric accuracy with which the tumor target can be localized with respect to the treatment beam. This challenge of target localization arises from organ motion and the difficulty of daily reproducibility of the planned position. It is the target localization uncertainty that usually dictates margin expansion from the clinical tumor volume (CTV) to the planning treatment volume (PTV) in order to assure full coverage. Often the CTV to PTV margin overlaps critical structures, and reduction of dose to prevent normal tissue toxicity is necessary at the expense of compromising tumor control (Balter J M et al. 1993). To improve it, on-line verification of target location was developed. The most prevalent one is 2D radiographic imaging techniques. Digital MV x-ray imaging using the treatment beam with an electronic portal imaging device (EPID) is one commonly used, though MV images have very poor soft tissue contrast (Bissett R et al. 1995, 1996). For better contrast the clinical KV imaging system was used in room-mounted configuration, such as, Cyberknife^®^ (Accuray Incorporated) and Novalis^®^ tracking system (BrainLAB AG). Phantom studies on those systems reveal both are capable of sub-millimeter accuracy (Yan H et al. 2003, Yu C et al. 2004). There are several gantry mounted systems such as the Elekta Synergy^®^ system (Elekta AB) and Varian On-Board Imager™ (Varian Medical Systems) for fast KV imaging.

It is desirable to employ 3D on-line tomography on patient positioning and target localization due to the capability of 3D volumetric matching of soft tissue. Certain clinical on-board imaging and CT reconstruction systems were developed in recent years (Jaffray D A et al. 1999; Jaffray D A and Siewerdsen J H, 2000; Jaffray D A et al. 2002; Oldham M et al. 2005; Yin F F et al. 2005). The potential of cone-beam CT as an online imaging guidance tool for radiation therapy was recognized in recent years (Simpson R G et al. 1982; Swindell W et al. 1983). The concept of a cone-beam CT system utilizes recently developed flat-panel detector technology (Jaffray D A et al. 1999; Jaffray D A and Siewerdsen J H 2000; Jaffray D A et al. 2002). These novel flat-panel detectors consist of an array of amorphous silicon (a-Si:H) based thin film transistors and photodiodes that operate in conjunction with a scintillating phosphor screen. The excellent optical coupling efficiency between the phosphor screen and a-Si based detector make it possible to produce quality images with less dose to the patient (Jaffray D A and Siewerdsen J H, 2000). These imagers could be read at ~30 frames per second allowing for the rapid acquisition of CBCT data (Jaffray D A and Siewerdsen J H, 2000). Furthermore, such detectors are available with an active area of up to about 40 × 40 cm^2^ providing a sufficiently large field of view. Recently, CBCT has been integrated on linear accelerators for image guided radiation therapy. Two examples of such a system are Elekta Synergy^®^ System and Varian Trilogy™ System. It is now possible to acquire a CBCT image of a patient in treatment position to be used for on-line target localization. The acquired CBCT image data could be directly registered with planning CT image data for patient positioning verification (Oldham M et al. 2005; Yin F F et al. 2005).

There are two essential clinical considerations for any localization device: 1) mechanical accuracy—clinical feasibility and 2) clinical efficacy. The first question is essential to determine whether this system could be used for clinical application and is mainly related to the device. The second question is related to how well this device could be used for different clinical tasks and is more related to the object of the imaging application. It is pertinent to address these two issues separately. The purpose of this study is to evaluate the mechanical accuracy of Varian on-board imager (OBI) specified for CBCT-guided target localization. We use an anthropomorphic pelvis phantom to simulate shifts in the three translational directions and rotation around three axes. Our experiment is designed to determine the accuracy of the detected shift and rotation given by 3D volumetric matching between the CBCT images with planning CT images. Also, we determine the residual error after repositioning based on the couch correction vector. We identify the residual error as the localization accuracy using CBCT. This is the first study on the target localization accuracy using Varian OBI system. The data used to generate our results was based on the measurements consisting of over 300 CBCT scans. Besides that, there are about 100 CBCT scans taken for initial phantom positioning and 200–300 CBCT scans taken but failed to meet our acceptance criterion due to improper setup of system parameters or wrong operation of devices.

## Materials and Methods

### On-Board Imager Syste

In this study cone-beam CT images were acquired with an On-Board Imager (OBI) system that was retrofitted on a Varian 21 EX Clinac (Varian Medical Systems, Palo Alto CA). The OBI system consists of a kV x-ray source (KVS) and an amorphous silicon based kV flat panel image detector (KVD) mounted on the gantry by two robotic arms (ExactArms™). The distance between x-ray source and isocenter of Linac is 100 cm and the distance between isocenter and kV detector is 50 cm. The detector incorporates a 512 × 512 active matrix of amorphous silicon (a-Si:H) thin film transistors and photodiodes that operate in conjunction with a scintillating phosphor layer. The active area of the detector has a dimension of 30 cm × 40 cm. A bowtie filter is mounted on the x-ray source in front of the kV beam to attenuate more x-ray to the thinner part of the body in cross-section. Use of the bowtie filter reduces patient dose, and reduces x-ray scatter thus improving image quality.

**Figure 1:**
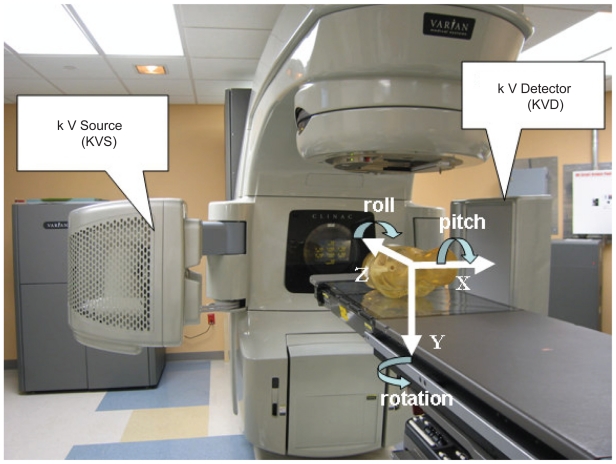
On-Board Imager system with coordinates.

The OBI system and treatment couch share the same coordinate system which is shown in Figure. 1 for an OBI system mounted on a Varian 21EX Clinac. The axis pointing towards the floor is the vertical axis (Y-axis). The downwards direction is defined as positive. The axis parallel to the treatment couch labeled Z is the longitudinal axis. Positive is defined as towards the gantry. The axis perpendicular to the Y-Z plane labeled X is the lateral axis. While facing the gantry, positive is on the right hand side and negative is on the left hand side. The rotation around the lateral axis (X axis) is called pitch. The rotation around the vertical axis (Y axis) is defined as rotation. The rotation around the longitudinal axis (Z axis) is called roll.

### CBCT Image Acquisition

In CBCT imaging mode the gantry makes over a full rotation while taking from 650–700 projection images. Scans could be acquired using a “half-fan” or “full-fan” acquisition mode depending on the object size. In our study, a “half-fan” acquisition mode is used to image the pelvis phantom. Because the OBI system was initially commissioned with the same criteria for both the “half-fan” and “full-fan” acquisition mode, we assume the geometric accuracy of the two acquisition modes should be the same. At our institution, CBCT scans on patients are performed with tube current set to 80 mA. We found it appropriate to use a low-dose setting (40 mA) for this study because contrast loss for our phantom using this dose setting had no effect on image registration. The main advantage in using the lower dose setting was a decrease in the amount of time required for cooling the housing between scans. The tube voltage was set to 125 kVp for all scans, and the 2-D dimensional projection data acquired from a CBCT scan was reconstructed into a 3D volumetric image by filtered back projection employing a Shepp-Logan convolution kernel and a modified Blackman Window convolution filter. For “half-fan” acquisition reconstructed pixel size was 0.89 mm × 0.89 mm. Approximately two minutes were required to complete the scan and the reconstruction.

### Image Registration

Varian’s OBI software contains 3D–3D Match analysis tools in which a user can register acquired CBCT images with reference CT images. Registration can be performed either manually or automatically mainly based on bony structures inside the phantom. In the manual match mode, the user is able to move the CBCT image relative to the stationary reference CT image in 6 degrees of freedom (i.e. x, y, z and rotation around each of those axes), to align the two sets of images. In the automatic-match mode, the software aligns the CBCT image with the CT reference image using an intensity-based registration method which relies on mutual information as a similarity measure. CBCT and CT images are aligned by isocenter, and an offset vector between them is determined. The registration algorithm shifts the CBCT image relative to CT reference image until the mutual information between the two datasets is maximized. Studies have shown that mutual information is a reliable tool for intensity based image registration (Kim J et al. 2001). It requires approximately one minute to perform an automatic registration using this feature.

After the two images are registered in either manual-match or automatic-match mode, the 3D displacement of the CBCT image relative to the reference CT image is given for the three translational directions and the three rotations around each of the translational axis (roll, pitch, and rotation). The couch shift parameter is the displacement vector required to align the target isocenter with the planned isocenter. They consist of the three translational directions and also rotation around the z-axis. The couch shift parameter vector could be represented by *V* as: *V = T_x_* + *T_y_* + *T_z_* + *R_z_* In this model *T_x,_* *T_x,_* *T_x,_* and *R_z_* represent displacement components of the lateral, longitudinal, and vertical directions and rotation around the Z-axis. The automatic-match mode was used for all registrations and followed by visual examination of match accuracy. If necessary, a manual match was performed. The couch shift parameter vector was then downloaded to the treatment machine to reposition the couch remotely.

### Experimental Design

**Figure 2:**
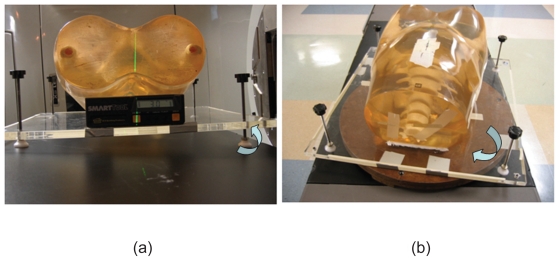
Techniques used to simulate a rotation of phantom (**a**) around the Z and X-axis, and (**b**) around the Y-axis.

**Figure 3:**
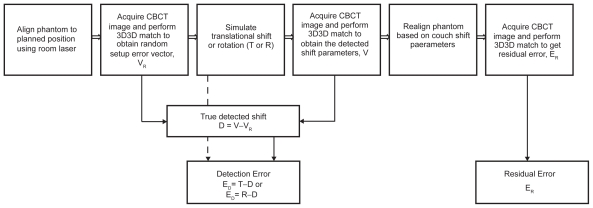
Flow chart showing experimental procedure.

A rigid pelvis anthropomorphic phantom was used to simulate an actual patient positioning scenario. CT planning data of the phantom were obtained with a GE Lightspeed RT unit (GE Medical Systems, Milwaukee, WI). The primary parameters of the protocol used in this phantom scan are 0.75 mm in pitch, 4 × 2.5 mm in detector size, and 1.0 second per gantry rotation. The scans were taken with tube current of 50 mA, and energy of 120 kV. Axial images were reconstructed with slice thickness of 2.5 mm which are commonly chosen for CT scanning of prostate patient. With the aid of the CT Simulator lasers, three metal fiducial markers were attached to the phantom on the anterior and both lateral surfaces marking the isocenter projected on the surface of the phantom. To enable rotation around the X and Z axis, the phantom was permanently attached to a ¾ inch thick plexi-glass platform using adhesive caulk as shown in Figure 2a. The platform has four height adjustable legs each at a corner. Two of the legs are raised at one time to produce a tilt. A SmartTool™ electronic level (1/10 degree accuracy) was used to determine the angle of the tilt. A rotational table was used to facilitate rotation around the Y-axis. This table was pre-marked with degree intervals used to measure the rotation. The techniques used to simulate a rotation are shown in Figure 3b. The flow chart in Figure 3 outline the experimental procedure for which each simulated translational shift or rotation was performed and evaluated. The aim of the experiment is to find the detection and residual error of target localization using CBCT.

**Figure 4:**
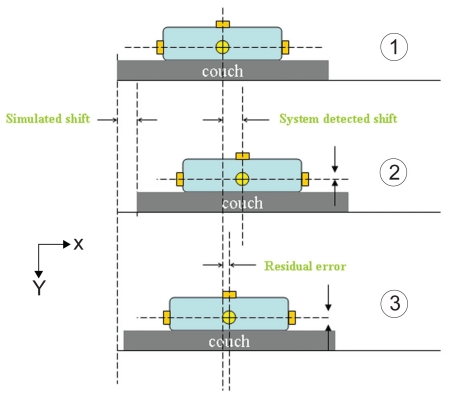
Experimental setup for a simulated translational shift.

The evaluation of the effect of translational shift on target localization accuracy consisted of the following steps as shown in Figure 4. In step one, the phantom was positioned on the treatment couch by aligning the fiducial markers with the room lasers. Afterwards, an initial CBCT scan was taken followed by 3D–3D matching. The detected shift vector at this point gives the random setup error of immobilizing the phantom to the planned position. In step two, a translational shift was simulated by moving the treatment couch a known direction and magnitude with assistance of laser. A second CBCT scan was followed by 3D–3D matching to yield another set of detected shift parameters. The setup error determined from the first scan is subtracted from the shift parameters of second scan. The result is compared to the known simulated shift to determine the localization accuracy of CBCT using OBI. In the third step, the couch shift vector was downloaded to the linac and the couch position was automatically shifted to realign with the isocenter of the phantom. Subsequently, to verify the accuracy of the repositioning a third CBCT scan was taken and 3D–3D matching performed. The detected shift vector from this scan indicates the residual error (repositioning error). For each translational direction, shifts of 1, 2, 5 and 10 mm were investigated in the method described above. To evaluate accuracy of the system in response to large shifts, longitudinal shifts of 20 and 50 mm were investigated. We did not investigate shifts larger than 50 mm due to potential hardware collision. The experiment for each combination of shift direction and magnitude was repeated five times.

**Figure 5:**
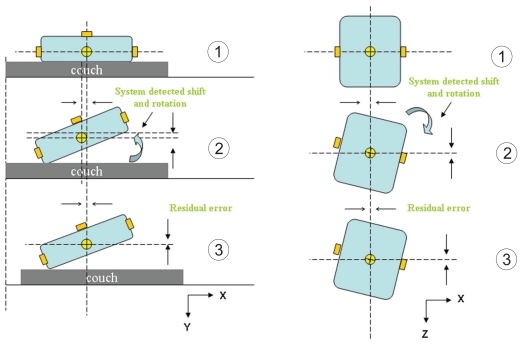
Experimental setup for a simulated rotation.

A similar procedure was used to investigate target localization of CBCT with respect to rotation as shown in Figure 5. Differing from procedure described above for evaluation of translational shift, the couch was fixed in step 2 and rotation was simulated by adjusting plexi-glass platform and rotational table as described earlier. Note that since the system cannot undo the rotation error due to the mechanical limitations of the couch and security reasons, the phantom was realigned to its planned position by a simple couch movement in three translational dimensions with the detected translational offsets. Simulated rotations around the X and Z-axis were evaluated for 1, 3 and 5 degrees by adjusting legs of glass platform. Simulated rotations around the Y-axis were evaluated for 3 and 5 degrees by adjusting angles of rotational table. Due to the large uncertainty associated with a rotation around the Y-axis, a 1 degree rotation was not evaluated. Also, rotations larger than 5 degrees were not investigated since after repositioning the new position of the couch could cause a potential hardware collision in subsequent scans. The experiment for each combination of rotation direction and magnitude was repeated five times.

The routine QA on geometrical and mechanical accuracy of LINAC, OBI, laser systems were conducted using calibration phantoms as the procedures reported by Sua Yoo, et al. and sub-millimeter accuracy can be maintained over a long period of time. The plexi-glass platform and rotational table were properly marked in facilitating the fast alignment with the laser in daily setup. Such alignment is reproducible within sub-millimeter accuracy. As the phantom employed in this study was rigid object and stick onto plexi-glass platform, the same position with respect to LINAC isocenter can be reproduced with high accuracy. It is noted that the initial scan taken at the first time of each test series represents the planned position of phantom plus minor daily setup error. To remove this daily setup error from each measurement, the shift of phantom detected from the first scan with respect to its planned position was subtracted from the shift of its location detected from subsequent scans to furnish the true shift of phantom as illustrated in Figure 3. All the experiments were conducted by a single operator to prevent operator-related variations. Each day 11 CBCT scans were conducted with one for initial reference scan and ten for five repetitive measurements in which two scans were conducted. If for any reason, the measurements could not be completed together, they were rescheduled for another day to avoid potential day-to-day setup errors.

### Data Analysis

For a translational shift, the detection error E_D_ was found from the calculation of T—D as shown in Figure 3. The vector T is the simulated translational shift. The vector D represents the detected shift without the contribution of the setup error. It is given by V—V_R_ where V is the detected offset vector obtained from the second CBCT scan and V_R_ is the random setup error vector obtained from the first CBCT scan. The residual error E_R_ is the couch shift vector obtained from the third CBCT scan. For the evaluation of translational shifts, only the translational components of the vectors described above were used in our calculations. For each combination of translational shift direction and magnitude, the detection error and residual error were found in each of five trials. The absolute value of that data was then used to find the average (*E*) and standard error (*SE*) based on the following equations:

(1) $$\bar{E}=\frac{1}{N}\sum_{i=1}^{N}E_{i},SE=\sqrt{\frac{\frac{1}{N-1}\sum_{i=1}^{N}{\left(E_{i}-\bar{E}\right)}^{2}}{\sqrt{N}}}$$

For a rotational offset, the detection error E_D_ was found from the calculation of R—D. The vector R is the simulated rotation around an axis. The vector D represents the detected offset without the contribution of the setup error. It is given by V—V_R,_ where V is the detected offset vector obtained from the second CBCT scan and V_R_ is the random setup error vector obtained from the first CBCT scan. Only the rotational components of the vectors were used for the calculation of the detection error. The residual error E_R_ is the couch shift vector obtained after the third CBCT scan. We report only the translational components of the couch shift parameters as the residual error. For each combination of rotation direction and magnitude, the detection error and residual error were found in each of five trials. The absolute value of that data was then used to find the average and standard error based on the Eq. 1.

## Results

On the average the detection errors and their standard deviations along the lateral, longitudinal and vertical directions are 0.3 ± 0.1, 0.3 ± 0.1 and 0.4 ± 0.1 mm respectively for translational shifts ranging from 0 to 10 mm. The corresponding residual error after positioning based on couch shift parameters are 0.3 ± 0.1, 0.5 ± 0.1 and 0.3 ± 0.1 mm. For longitudinal shifts of 20 mm and 50 mm, the average detection error along the lateral, longitudinal and vertical directions are 0.0 ± 0.0, 0.3 ± 0.2, 0.0 ± 0.0 mm respectively. The corresponding residual error are 0.0 ± 0.0, 0.6 ± 0.2, 0.1 ± 0.1 mm. The detection error in all directions ranges from 0.0 to 2.0 mm. The residual error ranges from 0.0 to 1.0 mm. The complete results are reported in Table 1. Each value of the table is an average based on five experimental trials. Also included is the standard error representing the estimated uncertainty of the reported values. The results show the relationship between shift magnitude with detection error and residual error for a giventranslational shift direction.

On the average the detection error and their standard deviation in pitch, roll, and rotation are 0.1 ± 0.0, 0.2 ± 0.0, and 0.2 ± 0.0 degrees respectively for simulated rotations ranging from 0 to 5 degrees. The residual errors after positioning based on couch shift parameters are 0.4 ± 0.1, 0.6 ± 0.1, and 0.3 ± 0.1 mm along the lateral, longitudinal and vertical directions. The detection error for all rotational directions ranged from 0 to 0.6 degrees. Their residual error ranged from 0 to 2 mm. The complete results are reported in Table 2. Each value of the table is an average based on five experimental trials. The given standard deviation estimates the value’s uncertainty. The results show the relationship between offset magnitude with detection error and residual error for a given rotational axis.

## Discussion

The outcome of our study indicates that using cone-beam CT for 3D target localization could achieve 1 mm accuracy as given by the detection error. We report this result as the maximum accuracy of the system since we used a rigid phantom in our study. The presence of a residual error is associated mainly with the limited accuracy of image registration and the mechanical resolution of the treatment couch. In an actual patient case, image registration will be less accurate compared to our rigid phantom scenario. One reason is patient anatomy could change between the time of the reference CT scan and the CBCT scan due to factors such as weight fluctuation, bowel filling, and deformation of organs and the tumor target. Thus, image registration would be performed between two images with inherent differences. Also, because of the relatively slow gantry speed of CBCT acquisition, respiratory motion could cause motion artifacts and blurring that greatly reduce image quality (Li T et al. 2006). Target localization using CBCT for non-rigid anatomic regions such as the pelvis, abdomen and thorax are expected to have less accurate results than our findings. However, those errors are related to patient and do not reflect the true capabilities of mechanical repositioning accuracy using CBCT.

We note there are uncertainties associated with our experimental method. For example, in our study of the effect of translational shift on localization accuracy, we applied shift by moving the treatment couch as it was practiced in routine clinical procedure. The mechanical resolution of the couch is 1 mm and thus the magnitude of the actual simulated shift could be within ± 0.5 mm of the intended magnitude. This contributes to the majority of residual error. It is expected that without the influence of couch shift uncertainty the smaller detection error can be achieved. As to the detection error, the uncertainty of couch movement is negligible because the phantom was always positioned with assistance of laser in addition to the couch motion. There are also uncertainties associated with our experiment on simulated rotations. The accuracy of producing a rotation around the X and Z axis (pitch and roll) is limited by our platform. For resolving this issue, we used an electronic level with 0.1 degree accuracy. Angular measurements need to be made on four sides of the platform when simulating a rotation of a desired magnitude. Considering that each measurement could have an error of 0.05 degrees, we estimate the maximum uncertainty of the simulated rotation to be 0.2 degrees. A rotation around the Y-axis (rotation) was based on degree markings on the rotational table which has an uncertainty of about 0.5 degrees. Considering the magnitude of detection errors achieved in this study, the variation caused by the uncertainty of rotation simulation has limited effect on the final accuracy of target localization. With the inclusion of uncertainty introduced by couch movement and rotation simulation, the average detection errors are 0.3 ± 0.6, 0.3 ± 0.6 and 0.4 ± 0.6 mm along the lateral, longitudinal and vertical directions with respect to translational shifts ranging from 0 to 10 mm, and 0.2 ± 0.2, 0.1 ± 0.2 and 0.2 ± 0.5 degrees in roll, pitch and rotation with respect to simulated rotations ranging from 0 to 5 degrees. The corresponding residual errors after repositioning are 0.3 ± 0.6, 0.5 ± 0.6 and 0.3 ± 0.6 mm with respect to translation shifts and 0.4 ± 0.6, 0.6 ± 0.6, and 0.3 ± 0.6 mm with respect to simulated rotations. Based on these detection errors and residual errors accounting for the uncertainty of measurement, the similar result was concluded that averagely the target can be identified within 1 millimeter and positioned within 2 millimeter using Varian OBI system.

In our study, both the CT reference images and CBCT images were reconstructed with a slice thickness of 2.5 mm. Each reconstructed slice of the CBCT image consists of 512 × 512 pixels with a pixel pitch of 0.89 mm. Despite the limited 2.5 mm resolution in cranial-caudal direction of both the CT and CBCT images, the registration algorithm was able to align the two images with better than 1 mm accuracy in all directions. This level of accuracy is attainable because the complete 3-D volumetric data sets are used in the registration. Yan et al. investigated the effect of CT slice thickness on target localization accuracy based on a real-time image guided system (Novalis^®^ Body system), and demonstrated that its influence on detection error is less for the slice plane and larger along the axial direction as slice thickness increased. Such an effect on accuracy would be expected in our system since it employs a similar image registration method. For the rotation experiments, we noticed using automatic matching was inaccurate in certain cases as evident from examining the matched images. For the cases in which automatic matching was inaccurate, we re-performed image registration using manual matching and recorded those corresponding data instead. The final result shows that some combination of rotation direction and magnitude has errors that are greater than others. However, there does not seem to be a trend in the variation. Recently, Yoo et al. showed that the OBI isocenter in the period of eight months, on the average deviated from the true isocenter by over half a millimeter in both the lateral and longitudinal direction (Yoo S et al. 2006). Based on their finding, we would expect that the isocenter of the OBI to vary throughout our experiment and thus we expect some variation in our results.

The overall result of our experiment demonstrates that CBCT is an accurate localization technique. However, its advantage of improved accuracy over traditional in-room localization methods should be weighed with its certain disadvantages. One of these disadvantages is the longer duration of the CBCT image acquisition process compared to traditional 2D radiographic imaging. This difference however is minimal and does not limit its clinical use. A much greater draw back is its imaging dose. Whereas the dose to acquire a set of orthogonal kV images is about 0.1 cGy, the dose of a CBCT is in the range of a few cGy (Islam M K et al. 2006). The dose of a single CBCT scan may be small compared to the treatment irradiation. However, in the course of a complete treatment its effect on normal tissue could be a concern.

## Conclusion

A phantom study was performed to evaluate the accuracy of using CBCT for target localization. We determined the detection error and residual error associated with translational shifts and rotation. The target localization accuracy is given by the detection error which is within the accuracy of sub-millimeter with respect to the simulated translational and rotation shifts in six degrees. In response to the offset detected by the CBCT based image registration, phantom was positioned based on treatment couch movement and averagely a sub-millimeter residual error is obtained. These results suggest the best accuracy which can be reached by Varian OBI system subjected to the software and mechanical limitations.

## Figures and Tables

**Table 1:** The average detection errors and residual errors associated with target localization after simulated translational shifts.

		Detection Error (mm)			Residual Error (mm)		

Shift Directions	Shift distances (mm)	Lateral	Longitudinal	Vertical	Lateral	Longitudinal	Vertical
Lateral (X)	1	0.0 ± 0.0	0.4 ± 0.2	0.0 ± 0.0	0.0 ± 0.0	1.0 ± 0.0	0.2 ± 0.2
	2	0.4 ± 0.2	0.8 ± 0.2	0.0 ± 0.0	0.4 ± 0.2	0.4 ± 0.2	0.0 ± 0.0
	5	0.1 ± 0.2	0.2 ± 0.0	0.0 ± 0.0	0.4 ± 0.2	0.4 ± 0.2	0.0 ± 0.0
	10	0.8 ± 0.2	0.2 ± 0.2	0.0 ± 0.2	1.0 ± 0.0	0.6 ± 0.2	0.4 ± 0.2
	1	0.2 ± 0.2	0.0 ± 0.0	0.4 ± 0.2	0.4 ± 0.2	0.4 ± 0.2	0.0 ± 0.0
	2	0.0 ± 0.0	0.0 ± 0.0	0.4 ± 0.2	0.0 ± 0.0	0.2 ± 0.2	0.0 ± 0.0
Longitudinal (Z)	5	0.0 ± 0.0	0.6 ± 0.2	0.6 ± 0.4	0.0 ± 0.2	0.8 ± 0.2	0.0 ± 0.0
	10	0.0 ± 0.0	0.6 ± 0.2	0.4 ± 0.2	0.2 ± 0.2	0.6 ± 0.2	0.0 ± 0.0
	20	0.0 ± 0.0	0.2 ± 0.4	0.0 ± 0.0	0.0 ± 0.0	0.4 ± 0.2	0.2 ± 0.2
	50	0.0 ± 0.0	0.0 ± 0.0	0.0 ± 0.0	0.0 ± 0.0	0.8 ± 0.2	0.0 ± 0.0
Vertical (Y)	1	0.6 ± 0.2	0.2 ± 0.2	0.4 ± 0.2	0.2 ± 0.2	0.2 ± 0.2	0.6 ± 0.2
	2	0.2 ± 0.2	0.2 ± 0.2	0.4 ± 0.2	0.6 ± 0.2	0.2 ± 0.2	1.0 ± 0.0
	5	0.4 ± 0.2	0.0 ± 0.0	0.8 ± 0.2	0.0 ± 0.0	0.2 ± 0.2	1.0 ± 0.0
	10	0.4 ± 0.2	0.2 ± 0.2	0.6 ± 0.2	0.2 ± 0.2	0.4 ± 0.2	0.8 ± 0.2

**Table 2:** The average detection errors and residual errors associated with target localization after simulated rotations.

		Detection Error (degrees)			Residual Error (mm)		

Rotation Directions	Rotation Magnitudes (degrees)	Roll	Pitch	Rotation	Lateral	Longitudinal	Vertical
	1	0.2 ± 0.0	0.0 ± 0.0	0.1 ± 0.0	0.0 ± 0.0	0.8 ± 0.2	0.0 ± 0.0
Roll	3	0.5 ± 0.0	0.0 ± 0.0	0.1 ± 0.0	0.2 ± 0.2	0.4 ± 0.2	0.0 ± 0.0
	5	0.1 ± 0.0	0.1 ± 0.0	0.1 ± 0.0	1.8 ± 0.2	0.2 ± 0.2	0.2 ± 0.2
	1	0.1 ± 0.0	0.0 ± 0.0	0.1 ± 0.0	0.2 ± 0.2	1.0 ± 0.0	0.2 ± 0.2
Pitch	3	0.1 ± 0.0	0.0 ± 0.0	0.4 ± 0.0	0.0 ± 0.0	1.6 ± 0.2	1.0 ± 0.0
	5	0.3 ± 0.0	0.0 ± 0.0	0.1 ± 0.0	0.4 ± 0.2	0.0 ± 0.0	0.2 ± 0.4
	3	0.0 ± 0.0	0.1 ± 0.0	0.1 ± 0.0	0.2 ± 0.2	0.8 ± 0.2	0.0 ± 0.0
Rotation	5	0.0 ± 0.0	0.0 ± 0.0	0.2 ± 0.1	0.0 ± 0.0	0.0 ± 0.0	0.0 ± 0.0
